# Distinct Olfactory Bulb-Cortex Neural Circuits Coordinate Cognitive Function in Parkinson’s Disease

**DOI:** 10.34133/research.0484

**Published:** 2024-10-02

**Authors:** Shuai-Shuai Wang, Xing-Feng Mao, Zhi-Shen Cai, Wen Lin, Xiu-Xiu Liu, Bei Luo, Xiang Chen, Yue Yue, Heng-Yu Fan, Takuya Sasaki, Kohji Fukunaga, Wen-Bin Zhang, Ying-Mei Lu, Feng Han

**Affiliations:** ^1^ Medical Basic Research Innovation Center for Cardiovascular and Cerebrovascular Diseases, Ministry of Education, China; International Joint Laboratory for Drug Target of Critical Illnesses, School of Pharmacy, Nanjing Medical University, Nanjing 211166, China.; ^2^ Department of Physiology, School of Basic Medical Sciences, Nanjing Medical University, Nanjing 211166, China.; ^3^ Department of Functional Neurosurgery, The Affiliated Brain Hospital of Nanjing Medical University, Nanjing 210029, China.; ^4^ Life Sciences Institute and Innovation Center for Cell Biology, Zhejiang University, Hangzhou 310058, China.; ^5^ Department of Pharmacology, Graduate School of Pharmaceutical Sciences, Tohoku University, Sendai, Japan.; ^6^ Key Laboratory of Modern Toxicology of Ministry of Education, Nanjing Medical University, Nanjing 211166, China.; ^7^ Gusu School, Nanjing Medical University, Suzhou Municipal Hospital, The Affiliated Suzhou Hospital of Nanjing Medical University, Suzhou 215009, China.; ^8^ Institute of Brain Science, the Affiliated Brain Hospital of Nanjing Medical University, Nanjing 210029, China.; ^9^ The Affiliated Huaian No.1 People’s Hospital of Nanjing Medical University, Northern Jiangsu Institute of Clinical Medicine, Huaian 223300, China.

## Abstract

Cognitive dysfunction stands as a prevalent and consequential non-motor manifestation in Parkinson’s disease (PD). Although dysfunction of the olfactory system has been recognized as an important predictor of cognitive decline, the exact mechanism by which aberrant olfactory circuits contribute to cognitive dysfunction in PD is unclear. Here, we provide the first evidence for abnormal functional connectivity across olfactory bulb (OB) and piriform cortex (PC) or entorhinal cortex (EC) by clinical fMRI, and dysfunction of neural coherence in the olfactory system in PD mice. Moreover, we discovered that 2 subpopulations of mitral/tufted (M/T) cells in OB projecting to anterior PC (aPC) and EC precisely mediated the process of cognitive memory respectively by neural coherence at specific frequencies in mice. In addition, the transcriptomic profiling analysis and functional genetic regulation analysis further revealed that biorientation defective 1 (*Bod1*) may play a pivotal role in encoding OB^M/T^-mediated cognitive function. We also verified that a new deep brain stimulation protocol in OB ameliorated the cognitive function of *Bod1*-deficient mice and PD mice. Together, aberrant coherent activity in the olfactory system can serve as a biomarker for assessing cognitive function and provide a candidate therapeutic target for the treatment of PD.

## Introduction

Cognitive impairment, as a non-motor symptom, is common in Parkinson’s disease (PD), but its mechanism is not fully understood [1]. In the early stage of PD, aggregation and misfolding of α-synuclein occur in the olfactory bulb (OB) [2], making olfactory impairment one of the earliest and uniform indication before the diagnosis of PD [3]. Until now, little is known about how the olfactory system is involved in the pathological process of cognitive impairment in PD.

The OB is a key part responsible for olfactory memory. Recent evidence reveals that the acquisition and formation of memory rely primarily on the neural connectivity between OB and piriform cortex (PC) [4,5]. Moreover, the OB is also connected with entorhinal cortex (EC)-dorsal hippocampal circuit, an area closely associated with recognition memory [6,7]. On the other hand, both EC and PC atrophy were detected in PD patients [8,9]. These indicate that OB, PC, and EC may cooperate to regulate cognitive function [10–13]. However, how the neural circuits among OB, PC, and EC are disrupted in the cognitive impairment of PD remains largely unclear.

The synchronization between different brain regions through frequency-specific connectivity is crucial for learning and memory [14–16]. Moreover, it has reported that beta-band oscillations in the cortico-striatal-thalamic circuit are enhanced to exert anti-kinetic functions in PD [17,18]. These studies inspire us to reveal the oscillatory characteristics of OB-related circuits, aiming to find out parameters for electro-treatments against cognitive impairment in PD patients.

Here, we provided the first evidence for abnormal functional connectivity (FC) among OB, PC, and EC in PD patients. We further uncovered that divergent M/T cells in the OB projecting to anterior PC (aPC) and EC differed in spatial distribution and electrophysiological characteristics in mice. Moreover, we clarified that the formation and retrieval of cognitive memory were encoded by neural coherences in OB^M/T^→aPC and OB^M/T^→EC circuits, but impaired in PD mice. Overall, these findings offer new clues for diagnosing and treating cognitive dysfunction in PD.

## Results

### Aberrant FC across the olfactory regions in PD patients and mice

**Fig. 1.**
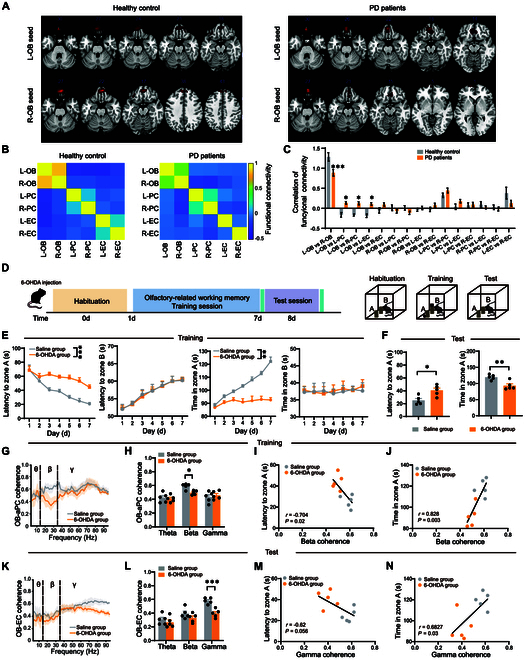
Aberrant functional connectivity (FC) across the olfactory regions in PD patients and mice. (A) Representative fMRI of the FC from olfactory bulb (OB) between PD patients and healthy control groups. (B) Heatmap of correlation of FC from OB with PC and EC. (C) Quantification of correlation of FC across olfactory circuits between PD patients (*n* = 12) and healthy control groups (*n* = 11). (D) Diagram of the olfactory-related working memory test. Before the test, mice are habituated to the experimental condition. In the training session, one cheese is hidden behind pole A, and the other with a metal mesh cover is placed on the top of pole B. In the test session, the cheese is removed from pole A and pole B. (E) Quantification of latency to zone A and B and total time in zone A and B during the training session between control and PD mice (*n* = 5 in each group). (F) Quantification of latency to zone A and total time in zone A and B during the test session between control and PD mice (*n* = 5 in each group). (G and H) OB-aPC coherence of LFPs in control and PD mice during the training session (*n* = 5 in each group). (I and J) Pearson’s correlation analysis of latency to zone A and total time in zone A during the test session versus the OB-aPC coherence at the beta band in the training session (*n* = 5 in each group). (K and L) OB-EC coherence of LFPs in control and PD mice during the test session (*n* = 5 in each group). (M and N) Pearson’s correlation analysis of latency to zone A and total time in zone A during the test session versus the OB-EC coherence at the gamma band in the test session (*n* = 5 in each group). Data are presented as mean ± SEM; ^**^P < 0.01, ^***^P < 0.001; ns, not significant. Two-way ANOVA for (C), (E), (H), and (I). Unpaired 2-tailed Student’s *t* test for (F).

Here, we investigated the FC strength within olfactory-associated brain regions in PD patients afflicted with cognitive dysfunction. Clinical functional magnetic resonance imaging (fMRI) results showed abnormal FC between OB and PC, and between OB and EC in PD patients with cognitive dysfunction (Fig. 1A to C).

To further elucidate the effects of aberrant FC on cognitive memory, an olfactory-related working memory test was performed to test the cognitive function in PD mice (Fig. 1D). Behavioral results indicated that PD mice displayed prolonged latency and reduced total exploration time in zone A compared to control mice during both training and test sessions (Fig. 1E and F). Next, we analyzed the coherence of local field potentials (LFPs) between the OB, aPC, and EC during the olfactory-related working memory test. OB-aPC coherence at the beta band was significantly lower during the training session (Fig. 1G and H), which is correlated to the latency and total exploration time in zone A (Fig. 1I and J), while OB-EC coherence did not change during the training session (Fig. S1A and B). Interestingly, during the test session, PD mice showed a decrease in OB-EC coherence at the gamma band (Fig. 1K and L), which is closely related to the total exploration time in zone A (Fig. 1M and N), with no change in OB-aPC coherence (Fig. S1C and D). In summary, the data above suggested that cognitive decline in PD was associated with aberrant FC in the olfactory regions, possibly induced by alterations in neural oscillatory coherences at specific frequencies.

### Anatomical distribution and electrophysiological characteristics of OB projections to the aPC and EC

**Fig. 2.**
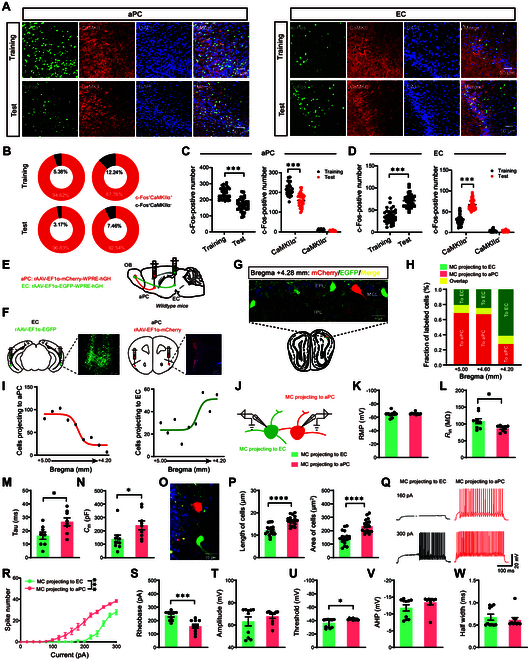
Anatomical distribution and specific electrophysiological characteristics of OB^M/T^ projections to the aPC and EC. (A) Representative confocal image of c-Fos (green) and CaMKIIα (red) in the aPC and EC during the olfactory-related working memory test. aPC, anterior piriform cortex; EC, entorhinal cortex. Green, c-Fos; red, CaMKIIα. Scale bars, 50 μm. (B) Pie chart of coexpression of c-Fos^+^, CaMKIIα^+^, and DAPI for (A). (C and D) Quantification of coexpression of c-Fos^+^ with DAPI (left) or with CaMKIIα^+^ (right) after the training or test session in the aPC (C) or EC (D) (*n* = 6 in each group, and 2 views of slices were provided for each mouse). (E and F) Schematic (E) and histology (F) of rAAV2/9-EF1α-mCherry-EGFP-hGHpA injection into the aPC and rAAV2/9-EF1α-EGFP-WPRE-hGHpA injection into the aPC and EC. (G) Representative image of coronary brain sections of mitral/tufted (M/T) cells in C57 mice after rAAV2/9-EF1α-mCherry-EGFP-hGHpA injection into the aPC and rAAV2/9-EF1α-EGFP-WPRE-hGHpA injection into the EC. (H) Fraction of mCherry^+^ (red), EGFP^+^ (green), and dually labeled (yellow) neurons in the OB as a function of bregma. (I) Distribution of OB-aPC (mCherry^+^) and OB-EC (EGFP^+^) M/T cells in the ipsilateral OB from bregma +5.00 mm to +4.20 mm. (J) rAAV-EF1α-mCherry-WPRE-hGH and rAAV-EF1α-EGFP-WPRE-hGH were injected in the aPC and EC, respectively, and whole-cell recordings were performed in mCherry^+^ or EGFP^+^ M/T cells in the OB. (K to N) Quantification of the intrinsic membrane properties in mCherry^+^ (*n* = 9 cells from 3 mice) or EGFP^+^ M/T cells (*n* = 10 cells from 3 mice). Resting membrane potential (RMP) (K), *R*_in_ (L), Tau (M), *C*_m_ (N). (O) Representative image of coronary brain sections of M/T cells in C57 mice after rAAV2/9-EF1α- mCherry-EGFP- hGHpA injection into the aPC and rAAV2/9-EF1α-EGFP-WPRE-hGHpA injection into the EC. (P) Quantification of the length and area of EGFP^+^ M/T cells or mCherry^+^ M/T cells (*n* = 19 cells from 3 mice). (Q) Representative AP firing of EGFP^+^ M/T cells and mCherry^+^ M/T cells evoked by current injections from 0 to 300 pA (stepped by 20 pA). (R and S) Quantification of the AP frequency (R) and current threshold of AP (S) of EGFP^+^ M/T cells and mCherry^+^ M/T cells evoked by current injections from 0 to 300 pA (stepped by 20 pA). (T to W) Quantification of the AP properties in EGFP^+^ M/T cells (*n* = 10 cells from 3 mice) or mCherry^+^ M/T cells (*n* = 9 cells from 3 mice). Black column, EGFP^+^ M/T cells; red column, mCherry^+^ M/T cells. AP amplitude (T), AP firing threshold (U), AP half-width (V), AP after-hyperpolarization (W) (*n* = 5 mice, unpaired 2-sample Student’s *t* test). Data are presented as mean ± SEM; ^*^P < 0.05, ^***^P < 0.001, ^****^P < 0.0001. Two-way ANOVA for (C) (right), (D) (right), and (R). Unpaired 2-tailed Student’s *t* test for (C) (left), (D) (left), (K) to (N), (P), and (S) to (W).

To further understand the precise role of the aPC and EC in olfactory-related working memory [19,20] , we first investigated the expression of c-Fos, which was found prominently expressed in CaMKIIα^+^ neurons in the aPC and EC during both training and test sessions (Fig. 2A and B). Moreover, the increase of c-Fos-positive cells was observed in aPC during the training session and in EC during the test session (Fig. 2C and D). These findings suggest that OB-aPC and OB-EC circuits may play distinct roles in the formation and retrieval of cognitive memory.

Considering that mitral/tufted (M/T) cells serve as the sole output neurons of OB [21], we labeled M/T cells projecting to the aPC and EC by injecting retrograde tracers, either rAAV-EF1α-mCherry-WPRE-hGHpA or rAAV-EF1α-EGFP-WPRE-hGHpA, into the aPC or EC, respectively (Fig. 2E and F). The image results showed that M/T cells labeled with enhanced green fluorescent protein (EGFP) or mCherry belonged to the 2 different subpopulations in the mitral cell layer of the OB (Fig. 2G and H). In the ipsilateral OB from bregma +5.00 mm to +4.20 mm, distribution of mCherry^+^ cells projecting to the aPC showed a descending trend, while the distribution of EGFP^+^ cells projecting to the EC showed an ascending trend (Fig. 2I). These observations suggested that M/T cells in the OB projecting to aPC (OB_aPC_) and EC (OB_EC_) were spatially segregated.

We further observed that the membrane properties of OB_aPC_ and OB_EC_ M/T cells were also different (Fig. 2J), including input resistance (*R*_in_), membrane time constants (*τ*), and membrane capacitance (*C*_m_) (Fig. 2K to N). As *C*_m_ and *R*_in_ are proportional to cell surface area and determine the membrane time constant, we further compared the length and surface area of OB_aPC_ and OB_EC_ M/T cells. The data indicated that the length and surface area of OB_aPC_ M/T cells were larger than those of OB_EC_ M/T cells (Fig. 2O and P). Moreover, OB_aPC_ M/T cells exhibited higher excitability [lower action potential (AP) threshold] compared to OB_EC_ M/T cells (Fig. 2Q to S). Other properties of AP were unaltered, including the amplitude, half-width, and after hyperpolarization (Fig. 2T to W). Taken together, these data showed that OB_aPC_ and OB_EC_ M/T cells differed in spatial and electrophysiological characteristics, which may have different roles on the formation and retrieval of cognitive memory.

### OB-EC coherence at the gamma band is required for the retrieval of cognitive memory

**Fig. 3.**
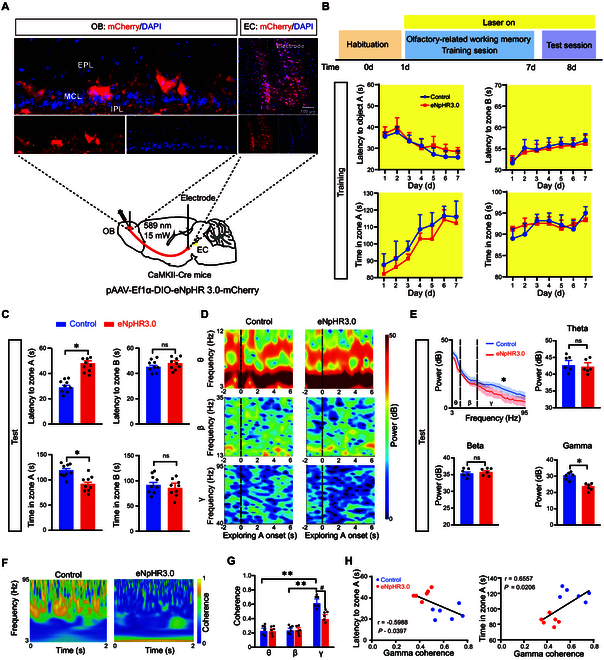
Coherence at the gamma band in the OB^M/T^→EC circuit monitors the retrieval of cognitive memory. (A) Schematic and histology of AAV-DIO-eNpHR3.0-mCherry or AAV-DIO-mCherry injection (as control) into the ventral OB and optical cannula implantation above the bilateral EC and single-electrode implantation in the OB and bilateral EC. DAPI was used as a nuclear marker. (B) Schematic of olfactory-related working memory test and quantification of latency to zone A or B and the total time in zone A or B during the training session (*n* = 9 in each group). (C) Quantification of latency to zone A and the total time in zone A during the test session (*n* = 9 in each group). (D) Relative power spectrum of LFPs recorded in the left EC around the onset of exploring zone A (*n* = 6 in each group). (E) Power spectrum of LFPs (3 to 95 Hz) in the EC during exploring zone A in the test session (*n* = 6 in each group). (F and G) Heatmap and quantification of OB-EC coherence of LFPs in control and eNpHR3.0 mice (*n* = 6 in each group). (H) Pearson’s correlation analysis of latency to zone A and total time in zone A during the test session versus OB-EC coherence at the gamma band. Data are presented as mean ± SEM; ^*^P < 0.05, ^**^P < 0.01, *^#^P* < 0.05. Two-way ANOVA for (B) and (H). Unpaired 2-tailed Student’s *t* test for (C) and (D).

To confirm our hypothesis, we further investigated the function of the OB^M/T^→EC circuit in olfactory-related working memory by stereotaxically injecting pAAV-Ef1α-DIO-eNpHR3.0-mCherry or pAAV-Ef1α-DIO-mCherry (as a control) into the ventral OB unilaterally, and implanted an optic fiber above the EC (Fig. 3A). Olfactory-related working memory behavioral tests were conducted after 3 weeks. Continuous yellow light was applied in EC to selectively inhibit the axonal terminals of M/T cells for 10 min (589 nm, 15 mW), We found that inhibiting the OB→EC circuit did not affect exploration behavior and synchronized LFP signals in EC during the training session (Fig. 3B and Fig. S2A). Additionally, there was no effect on the OB-EC coherence in the training session (Fig. S2B). However, significantly increased latency and decreased total exploration time in zone A were observed during the test session (Fig. 3C). Consistently, LFP signals revealed that optogenetic inhibition of the OB^M/T^→EC circuit attenuated gamma oscillation in the EC during the test session (Fig. 3D). Theta (3 to 12 Hz) and beta (13 to 35 Hz) oscillations were unaffected (Fig. 3E). Moreover, optogenetic inhibition of OB^M/T^→EC circuit reduced the OB-EC coherence at the gamma band (Fig. 3F and G), which was correlated with the latency of exploration and the total exploration time in zone A (Fig. 3H). Together, the data indicated that the OB-EC coherence at the gamma band may mediate the retrieval of cognitive memory.

### OB-aPC coherence at the beta band is required for the formation of cognitive memory

**Fig. 4.**
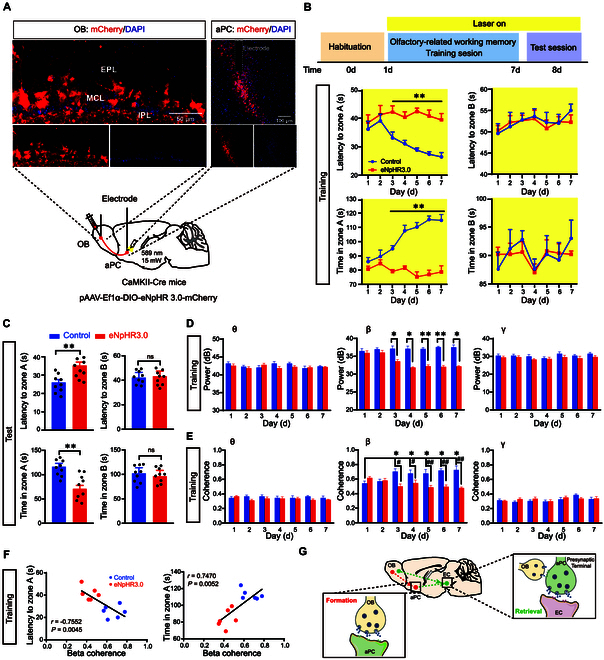
Coherence at the beta band in the OB^M/T^→aPC circuit adjusts the formation of cognitive memory. (A) Schematic and histology of pAAV-DIO-eNpHR3.0-mCherry or pAAV-DIO-mCherry (as control) injection into the ventral OB, optical cannula implantation in the bilateral aPC, and single-electrode implantation in the OB and bilateral aPC. (B) Schematic of olfactory-related working memory test and quantification of latency to zone A or B and total time in zone A or B during the training session (*n* = 9 in each group). (C) Quantification of latency to zone A or B and total time in zone A or B during the test session (*n* = 9 in each group). (D) Quantification of LFP power (3 to 95 Hz) in the aPC during exploring the zone A in the training session (*n* = 9 in each group). (E) Quantification of the OB-aPC LFP coherence (*n* = 9 in each group). (F) Pearson’s correlation analysis of latency and total time versus OB-aPC coherence at the beta band in the training session. (G) Schematic summarizing microcircuit of OB^M/T^→EC and aPC-EC projections in EC. Data are presented as mean ± SEM; ^*^P < 0.05, ^**^P < 0.01, *^#^P* < 0.05, *^##^P* < 0.01. Two-way ANOVA for (B), (D), and (F). Unpaired 2-tailed Student’s *t* test for (C).

Next, we inhibited the OB-aPC circuit by stereotaxically injecting pAAV-Ef1α-DIO-eNpHR3.0-mCherry or pAAV-Ef1α-DIO-mCherry (as a control) into the ventral OB unilaterally. Additionally, an optic fiber was implanted above the aPC (Fig. 4A). Three weeks after the surgery, we conducted the same behavioral experiments as shown in Fig 3 (Fig. 4B). Continuous yellow light was applied in aPC to selectively inhibit the axonal terminals of M/T cells for 10 min (589 nm, 15 mW). We observed that optogenetic inhibition of the OB-aPC circuit increased the latency and decreased the total exploration time to zone A during the training session (Fig. 4B), and also had a similar effect in the test session (Fig. 4C).

LFP recordings in the aPC showed that optogenetic inhibition of the OB^M/T^→aPC circuit decreased beta, but not theta and gamma oscillations from day 3 to day 7 during the training session (Fig. 4D), while no changes were observed in the aPC during the test session (Fig. S3A). Moreover, LFP recordings in the OB and aPC revealed that the OB-aPC coherence at the beta band was also enhanced by training in control mice, and this enhancing effect was weakened by optogenetic inhibition of the OB-aPC circuit (Fig. 4E), which was significantly correlated with the latency and total exploration time in zone A (Fig. 4F). In the test session, no significant difference in neural OB-aPC coherence was observed (Fig. S3B). These data suggested that the OB-aPC coherence at the beta band was required for the formation of cognitive memory.

As we expected, optogenetic inhibition of the OB^M/T^→aPC circuit increased the latency and decreased the total exploration time in zone A (Fig. 4C); however, there were no changes in aPC beta oscillation in the test session. Therefore, to investigate how optogenetic inhibition of the OB^M/T^→aPC circuit impaired behavioral performance during the test session, we injected retrograde tracking virus (rAAV2-Ef1α-EGFP-WPRE-hGHpA) into the EC, and the EGFP^+^ neurons were observed in the aPC 3 weeks after injection (Fig. S3C). Next, to further explore whether neural coherence between the aPC and EC is required for cognitive memory in the test session, we simultaneously recorded LFPs of aPC and EC when the OB^M/T^→aPC circuit was optogenetically inhibited. The results showed that gamma oscillations were highly correlated in the retrieval of cognitive memory, which was reduced by optogenetic inhibition of OB^M/T^→aPC circuit (Fig. S3D). Together, the data indicated that aPC, receiving direct inputs from OB, served as a pivotal hub for cognitive memory, which functioned in the formation process; meanwhile, its outputs to EC functioned in the retrieval process, which was modulated by OB→EC projection (Fig. 4G).

### *Bod1* deficiency induces decreased M/T cell activity

**Fig. 5.**
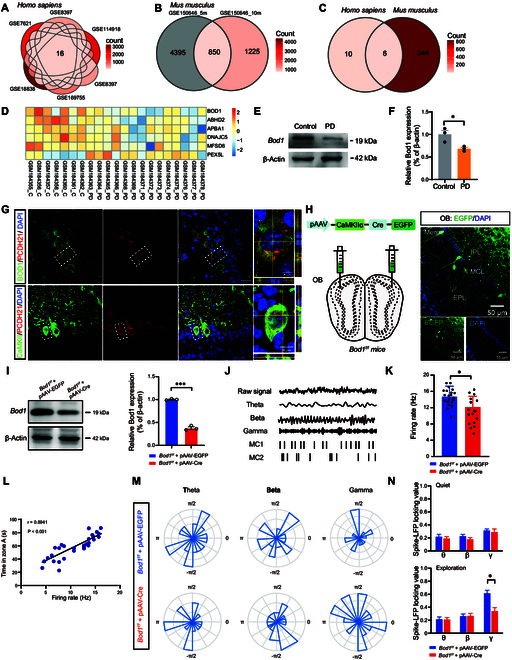
*Bod1* deficiency induces decreased M/T cell activity. (A to C) Overlapping Venn diagram of differentially expressed (DE) transcriptomic genes in 6 candidate gene databases of PD patients and 2 candidate gene databases of PD model mice. (D) Heatmap of changes in expression of 6 overlapping DE genes. (E and F) Representative band (E) and quantification (F) of BOD1 protein in the OB of PD mice (*n* = 3 in each group). (G) Representative images of immunofluorescence of BOD1, PCDH21, and CaMKIIα in the mitral cell layer (MCL) of the OB. DAPI mark for nucleus. Scale bars, 50 μm. (H) Schematic illustration of AAV-CaMKIIα-GFP-Cre injection into the mitral cell layer of *Bod1^f/f^* mice (left) and representative images of EGFP^+^ cells in the MCL of the OB after the injection of AAV-CaMKIIα-Cre-EGFP (right). Scale bars, 50 μm. (I) Representative band and quantification of Bod1 protein in the OB of *Bod1^f/f^* mice and *Bod1*-deficient mice (*n* = 3 in each group). (J) Examples and criteria for spiking-LFP phase-locking raw data. Raw LFPs, bandpass-filtered LFPs, and representative raster plots of 2 M/T cells (MC1 and MC2) recorded simultaneously in the OB. (K) Quantification of the mean firing rate in *Bod1^f/f^* and *Bod1-*deficient mice during the exploring state. (L) Correlation between firing rate and total time spent exploring zone A (*n* = 14 in each group). (M) Representative circular distribution of mean spike locking to theta, beta, and gamma phase angles for the 2 M/T cells in (J). (N) Ratio of M/T cells locked to a given frequency band during the quiet (left) and exploration (right) states (*n* = 21cells in *Bod1^f/f^* mice, *n* = 18 cells in *Bod1-*deficient mice). Data are presented as mean ± SEM; ^*^P < 0.05. Two-way ANOVA for (N). Unpaired 2-tailed Student’s *t* test for (F), (I), and (K).

To investigate the potential molecular mechanism underlying cognitive decline mediated by abnormal neural coherence across olfactory circuits, transcriptomic profile datasets from clinical PD patients and PD model mice were preformed, and we observed that *Bod1* was significantly down-regulated compared to control (Fig. 5A to D). Further analysis showed that the level of Bod1 in OB of PD mice was significantly decreased (Fig. 5E and F). The immunofluorescence staining images showed that almost all protocadherin 21 (PCDH21), which is highly restricted to M/T cells [22], were colocalized with *Bod1* (Fig. 5G). We also found that CaMKIIα^+^ neurons were almost colocalized with PCDH21 (Fig. 5G). To further reveal the function of *Bod1* on M/T cells, we injected pAAV-CAMKIIα-GFP-2A-Cre (as *Bod1*-deficient mice) or pAAV-CAMKIIα-MCS-EGFP-3FLAG (as a control) into the mitral cell layer in the OB of *Bod1^f/f^* mice (Fig. 5H) and found that the protein level of Bod1 significantly decreased (Fig. 5I). The phase-locked value between spikes and LFPs was evaluated to reflect the relationship between the LFP fluctuations and single-neuron responses. We recorded neuronal oscillations by implanting electrodes into the mitral cell layer of the OB, and single unit of M/T cells was isolated [23]. We found a significant decrease in the firing rates of M/T cells in *Bod1*-deficient mice (Fig. 5J and K), with an obvious positive correlation between the firing rate of M/T cells and behavior (total exploration time in zone A) (Fig. 5L). Moreover, during olfactory exploration, we found that compared to control mice, *Bod1*-deficient mice exhibited a lower locking probability in the gamma band in the exploration state, while there was no significant difference in the quiet state (Fig. 5M and N). Together, these data indicated that *Bod1* deficiency disrupted functional encoding through offset discharges in the OB.

### *Bod1* deficiency leads to deficits in cognitive memory mediated by aberrant neural coherence

**Fig. 6.**
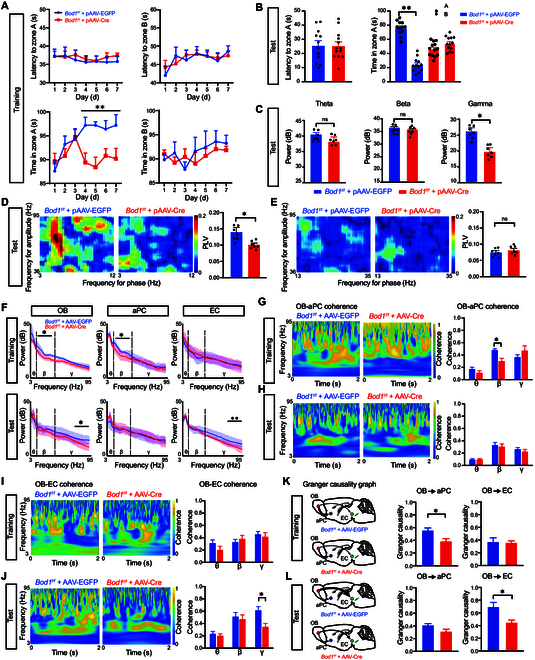
*Bod1* deficiency in M/T cells induces cognitive memory deficits by abating oscillatory activity in the OB and neural coherence across OB^M/T^→aPC and OB^M/T^→EC circuits. (A and B) Quantification of latency to zone A and B and total time in zone A and B during the training (A) and test sessions (B) in *Bod1^f/f^* (*n* = 12) and *Bod1-*deficient mice (*n* = 13). (C) Representative raw LFPs, bandpass-filtered LFPs, and quantification of the powers of the theta, beta, and gamma bands during the test session (*n* = 7 in each group). (D and E) Overall standard PAC comodulogram of the phase locking value (PLV) for theta phase–gamma amplitude coupling (D) or beta phase–gamma amplitude coupling (E) between *Bod1^f/f^* and *Bod1-*deficient mice across the test session (*n* = 7 in each group). (F) Power spectrum of LFPs in *Bod1-*deficient mice or *Bod1^f/f^* mice during the training and test session. LFP recording in the OB, aPC, and EC simultaneously in the olfactory-related working memory test. (G and H) Quantification of the OB-aPC coherence of LFPs in *Bod1-*deficient mice or *Bod1^f/f^* mice in the training (G) and test session (H) (*n* = 7 in each group). (I and J) Quantification of the OB-EC coherence of LFPs in *Bod1-*deficient mice or *Bod1^f/f^* mice during the training (I) and test session (J) (*n* = 7 in each group). (K and L) Spectral Granger causality averaged over OB-aPC and OB-EC electrode pairs in *Bod1-*deficient mice or *Bod1^f/f^* mice during the training session (K) and test session (L) (*n* = 7 in each group). Data are presented as mean ± SEM; ^*^P < 0.05, ^**^P < 0.01. Two-way ANOVA for (A), (B) (right), and (G) to (J). Unpaired 2-tailed Student’s *t* test for (B) (left), (C) to (E), (K), and (L).

To further investigate the effect of *Bod1* deficiency on cognitive memory, the olfactory-related working memory test was performed as described above. In the training session, the control mice spent more time in zone A than did the *Bod1*-deficient mice, and this trend disappeared in zone B (Fig. 6A). Simultaneously, in the test session, the control mice spent more time in zone A than in zone B, which disappeared in *Bod1*-deficient mice (Fig. 6B). However, the 2 groups showed no difference in latency to zone A.

Next, we investigated the role of *Bod1* in neuronal oscillations by implanting electrodes into the mitral cell layer of the OB. The data displayed that in the test session, the power at the gamma band in *Bod1*-deficient mice was significantly decreased, but no change in theta and beta bands (Fig. 6C). We employed standard phase-amplitude coupling (PAC) algorithms to assess the modulation of gamma oscillations by theta oscillations, and found a significant decrease in coupling strength between gamma amplitude and theta phase in *Bod1*-deficient mice during the test session (Fig. 6D), and there was no significant difference in coupling between the gamma amplitude and the beta phase compared with control mice (Fig. 6E).

To explore how *Bod1* deficiency in OB^M/T^ affects the neural oscillation activities in the olfactory-related working memory test, we simultaneously recorded the LFPs in the 3 brain regions by implanting single electrodes in the OB, aPC, and EC. The power spectral density analysis showed that *Bod1* deficiency attenuated beta-band activity in the OB and aPC during the training session, but no changes were observed in the EC. Then, during the test session, *Bod1* deficiency attenuated gamma-band activity in the OB and EC, with no change in the aPC (Fig. 6F).

We further analyzed the OB-aPC and OB-EC coherences, and found that *Bod1* deficiency dampened OB-aPC coherence at the beta band in the training session (Fig. 6G), but had no effect on OB-EC coherence (Fig. 6I). Conversely, there was no significant difference in OB-aPC coherence in the test session (Fig. 6H), while *Bod1* deficiency abated OB-EC coherence at the gamma band (Fig. 6J). In addition, Granger causality spectrum analysis was used to explore the role of beta and gamma oscillations in the causal connectivity between OB^M/T^→aPC and OB^M/T^→EC circuits. During the training session, *Bod1*-deficient mice displayed a significantly reduced Granger causality index in the beta band, specifically in the direction from the OB to aPC circuit, while no alterations were observed in the direction from the OB to EC circuit compared to control mice (Fig. 6K). In the test session, *Bod1*-deficient mice exhibited a significantly decreased Granger causality index at the gamma band in the direction from the OB to EC circuit, with no changes noted in the OB to aPC circuit compared to control mice (Fig. 6L).

The data suggested that *Bod1* deficiency in M/T cells induced the formation and retrieval of cognitive memory deficits by abating oscillatory activity in the OB and neural coherence across OB^M/T^→aPC and OB^M/T^→EC circuits.

### HFS ameliorates the cognitive memory deficits of *Bod1*-deficient and PD mice

**Fig. 7.**
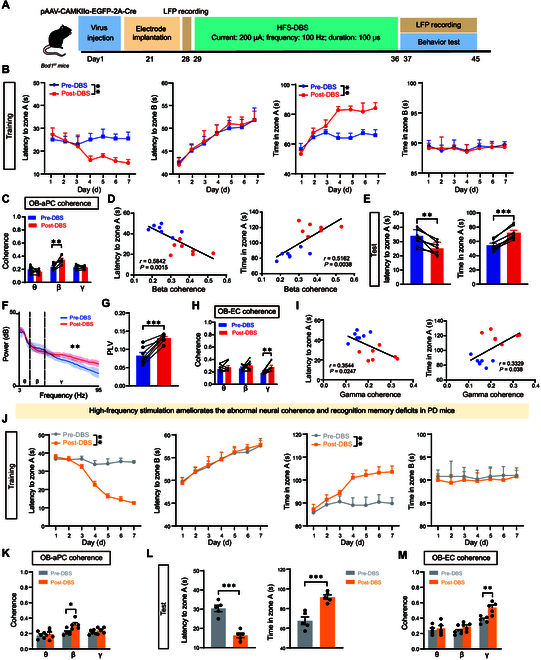
High-frequency stimulation (HFS) of OB ameliorates the abnormal neural coherences and cognitive memory deficits in *Bod1*-deficient mice and PD mice. (A) Schematic of HFS-DBS combined with olfactory-related working memory test. HFS (200 μA; 100 Hz; duration: 100 μs) to the OB in *Bod1*-deficient mice and PD mice. (B) Quantification of latency to zone A and B and total time in zone A and B during the training session between pre- and post-HFS DBS in *Bod1-*deficient mice (*n* = 7). (C) OB-aPC coherence of LFPs in pre- and post-HFS DBS in *Bod1-*deficient mice during the training session (*n* = 7). (D) Pearson’s correlation analysis of latency to zone A and total time in zone A during the test session versus the OB-aPC coherence at the beta band in the training session. (E) Quantification of latency to zone A and total time in zone A and B during the test session between pre- and post-HFS DBS in *Bod1-*deficient mice (*n* = 7). (F) Power spectrum of LFPs in the OB pre- and post-HFS-DBS in *Bod1-*deficient mice. (G) PLV for theta phase–gamma amplitude coupling pre- and post-HFS DBS in *Bod1-*deficient mice during the test session (*n* = 7). (H) OB-EC coherence of LFPs in pre- and post-HFS DBS in *Bod1-*deficient mice during the test session (*n* = 7). (I) Pearson’s correlation analysis of latency to zone A and total time in zone A during the test session versus the OB-EC coherence at the gamma band in the test session (*n* = 7). (J) Quantification of latency to zone A and B and total time in zone A and B during the training session between pre- and post-HFS DBS in PD mice (*n* = 5). (K) OB-aPC coherence of LFPs in pre- and post-HFS DBS in PD mice during the training session (*n* = 5). (L) Quantification of latency to zone A and total time in zone A and B during the test session between pre- and post-HFS DBS in PD mice (*n* = 5). (M) OB-EC coherence of LFPs in pre- and post-HFS DBS in in PD mice during the test session (*n* = 5). Data are presented as mean ± SEM; ^**^P < 0.01, ^***^P < 0.001. Two-way ANOVA for (B), (C), (H), (J), (K), and (M). Paired *t* test for (E), (G), and (L).

Deep brain stimulation (DBS) is used to treat intractable brain diseases, including PD, dystonia, and essential tremors [24,25]. To explore whether DBS could improve the cognitive memory deficits induced by abnormal oscillatory activity due to *Bod1* deficiency in the OB^M/T^, we administrated high-frequency stimulation (HFS) (200 μA, 100 Hz, 100 μs) or low-frequency stimulation (LFS) (200 μA, 10 Hz, 100 μs) to specifically modulate neural activity patterns in the OB. First, we recorded the LFPs in the OB, aPC, and EC regions as baseline (pre-DBS). Electrical stimulation was given 30 min/day for 7 d, following the olfactory-related working memory test was performed and LFP signal was recorded in the OB, aPC, and EC regions (Fig. 7A). The results showed that HFS treatments shortened the latency and increased the total exploration time in the training session (Fig. 7B). We also analyzed the coherences of the LFPs across the 3 brain regions. The results showed that HFS ameliorated OB-aPC coherence at the beta band (Fig. 7C). Furthermore, we assessed that the behavior improvement of *Bod1*-deficient mice was significantly correlated with the increased OB-aPC coherence at the beta band by HFS (Fig. 7D).

In the test session, HFS treatment also caused *Bod1*-deficient mice to spend less latency and more total exploration time in zone A (Fig. 7E). The power spectral density analysis suggested that HFS increased the power of gamma oscillation, but not beta and theta oscillations in OB, and the gamma amplitude–theta phase coupling was also strengthened (Fig. 7F and G). Furthermore, HFS treatment ameliorated OB-EC coherence at the gamma band (Fig. 7H), which was significantly correlated with the shortened latency and the increased total exploration time in zone A (Fig. 7I). However, LFS treatment had no effects on cognitive memory behavior analysis and LFP signal in both training and test sessions (Fig. S4).

Furthermore, we investigated the effect of HFS treatment in PD mice. In the training session, HFS treatments shortened the latency and increased the total exploration time in zone A in PD mice (Fig. 7J), which was accompanied by ameliorated OB-aPC coherence at the beta band (Fig. 7K). In the test session, HFS treatments also caused PD mice to spend less latency and more total exploration time in zone A (Fig. 7L), along with ameliorated OB-EC coherence at the gamma band (Fig. 7M).

Taken together, these data indicated that HFS could reverse the abnormal oscillatory activity and ameliorate the cognitive memory deficits induced by the specific deletion of *Bod1* in the OB^M/T^.

## Discussion

**Fig. 8.**
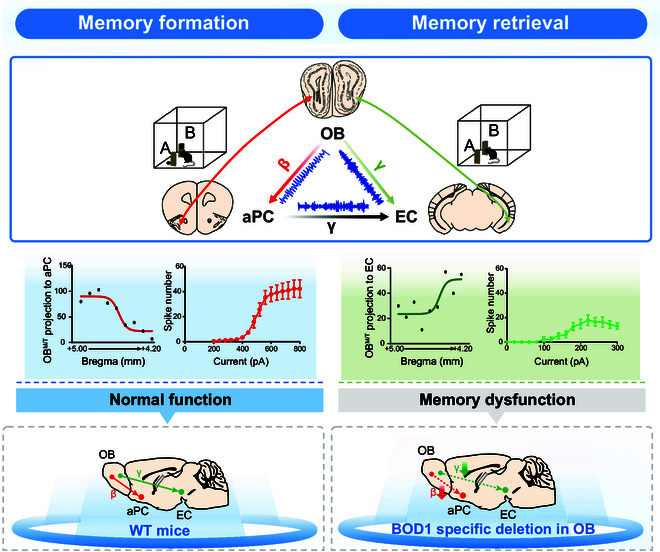
Schematic summary of distinct OB-cortex neural circuits that coordinate cognitive function. Top: OB projecting to aPC and EC precisely mediated the process of cognitive memory by neural coherence at specific frequencies in mice. Middle: 2 populations of projecting M/T cells to aPC and EC in the OB showed different spatial distribution and electrophysiological activity. Bottom: Bod1-deficient mice showed abnormal neuronal oscillation coherence in the OB^M/T^→aPC and OB^M/T^→EC circuits, which mediated cognitive impairment.

A clearer understanding of how olfactory circuit perturbations impair cognitive function in PD can lay a foundation for identifying predictors and solvers of PD. Here, our clinical fMRI analysis revealed that OB-PC and OB-EC coherences decreased in PD patients with cognitive dysfunction. Specifically, we uncovered that *Bod1* played a mechanistic role in neuronal oscillation coherence in the OB^M/T^→aPC and OB^M/T^→EC circuits, in which the 2 populations of projecting M/T cells in the OB showed different spatial distribution and electrophysiological activity. Hence, our findings elucidate the neural oscillation coherence and molecular mechanism of OB dysfunction-mediated cognitive impairment in PD (Fig. 8).

How does oscillatory coherence in olfactory circuits orchestrate cognitive functions? To address this question, we first examined the large-scale neural oscillations among the OB, aPC, and EC during memory. In the present study, PD mice displayed decreases in OB-EC coherence at the gamma band and OB-aPC coherence at the beta band. Neural circuits coordinate the execution of organized cognitive function, whereas this coordination in local and remote neural circuits is disrupted in patients with PD, thereby contributing to cognitive impairment [26–28]. Accumulating data have revealed that frequency-domain consistency or phase synchronization is disturbed in both the beta and gamma bands between the OB and aPC in APP/PS1 mice [29]. However, it remains unexplained whether these oscillations directly damage cognition. Combined with optogenetic analysis, our study confirmed a direct relevance between OB-aPC oscillatory coherence and cognitive function. Theta-gamma coupling is essential for accurate coordination between the hippocampus and EC during encoding and retrieval of memory [30]. However, experimental evidence about how OB regulates the EC-associated cognitive network is still lacking. In our study, we found that optogenetic inhibition on OB^M/T^→EC circuit reduced the OB-EC coherence at the gamma band and disrupted the retrieval of cognitive memory, which corroborates recent studies by Chen et al. [31], who observed that OB activity is indispensable for functional lateral entorhinal cortex (LEC)-associated cognitive functions. Based on this, an electrophysiological mechanism may be proposed where cognitive decline in PD is mediated by OB-derived neural oscillatory coherence.

Furthermore, we defined the molecular basis of neural coherence that is essential for formation and retrieval of cognitive memory. Synaptic plasticity and smooth interactions between different brain regions through neural circuits are preconditions for the encoding of cognitive memory. Previous studies have revealed that *Bod1* is involved in the processes of dendrite morphogenesis [32] and development of cognitive features [33–35]. Our study supports that *Bod1* is engaged in an evolutionarily conserved mechanism crucial for the development of cognitive features. Here, *Bod1* was highly expressed in the M/T cells of the OB from Genotype-Tissue Expression and immunofluorescence data. We also found that *Bod1* deficiency dampened the firing rates of M/T cells and olfactory-associated cognitive memory in the OB. Moreover, *Bod1*-deficient mice expressed a low locking probability of M/T cells firing in the gamma band in the OB. Therefore, we speculate that *Bod1*, which is located at synapses in neurons, may act on neuronal firing and oscillatory coherence by modulating the number and shape of dendritic spines. Synaptic plasticity is also favored by the phase-locked relationship across neuronal networks [36–38]. We postulate that the weakened gamma frequency phase-locking of single neurons may explain *Bod1* deficiency-mediated changes in spine and dendrite morphogenesis, but further study is needed to reveal dynamic correlation between *Bod1* and neural oscillation coherence in the progression of PD.

Given the therapeutic potential of DBS against PD, specific brain regions, such as the globus pallidus, can be subjected to DBS for relieving motor symptoms [39–41]. Then, it would be of great value to understand the effect and target brain areas of DBS in non-motor symptoms of PD. Here, we found that DBS could reduce the abnormal oscillatory activity and cognitive memory deficit in *Bod1-*deficient and PD mice. Consistent with previous findings that HFS of anterior nucleus thalamus can correct impaired cognitive function [42], our study further explored the role of HFS in network oscillatory coherence. Interestingly, HFS (200μA stimulation current, 100Hz pulse frequency, 100μs duration) to the mitral cell layer of the OB significantly increased the OB-aPC coherence at the beta band in the training session and OB-EC coherence at the gamma band in the test session. Consistently, HFS inhibited cognitive decline in *Bod1-*deficient and PD mice. Our findings agreed with recent studies reporting that OB stimulation can optimize brain oscillation and enhance FC to prevent Alzheimer's disease-impaired working memory [43]. Simultaneously, our study further clarified the function of oscillatory coherence at different stages of cognitive memory.

To conclude, this study uncovers that the formation and retrieval of cognitive memory are mediated by different projections of OB^M/T^ to aPC and EC via frequency-specific coherence. Frequency-specific coherence and *Bod1* signaling are closely involved in the abnormal FC of olfactory circuit during the development of PD.

## Materials and Methods

### Animals

*Bod1^f/f^* mouse was generated by Nanjing BioMedical Research Institute of Nanjing University (NBRI) [32]. *CaMKII*-*Cre* mice were obtained from The Jackson Laboratory (stock no. 007914), and C57BL/6J mice were obtained from SHANGHAI SLAC (Shanghai, China). Male mice aged 6 to 8 weeks were utilized for the experiments. All mice were housed under standard conditions at 22 to 23 °C with a 12:12-h light/dark cycle and given food and water ad libitum. The genotype of *Bod1^f/f^* mouse line was confirmed by polymerase chain reaction (PCR). The primers are as follows: forward: 5′-CTGTCAGCTACAGGCTGCTG-3′ and reverse: 5′-TGACCTTCTCTCCAACTGGAGG-3′.

### Participants

All participants were recruited from the Brain Hospital Affiliated to Nanjing Medical University. A total of 37 PD patients and 13 healthy controls were included in the study, and all participants were right-handed. PD patients were diagnosed based on the United Kingdom Parkinson's Disease Society Brain Bank clinical diagnostic criteria [44] and Movement Disorder Society (MDS) clinical diagnostic criteria for PD [45]. Patients with concomitant central nervous system disease, taking medications affecting brain function (antipsychotics), or with any MRI contraindications were excluded.

### Image data acquisition, preprocessing, and functional connection analysis

Structural and functional imaging data were obtained from all the participants on a 1.5-T MRI scanner (GE Medical System) equipped with an 8-channel head coil. Functional images were obtained using an echo planar imaging (EPI) sequence using the following parameters: repetition time (TR) = 2,000 ms, echo time (TE) = 40 ms, flip angle (FA) = 90°, thickness = 3.0 mm with no gap, number of slices = 28, matrix size = 64 × 64, field of view (FOV) = 240 mm × 240 mm, voxel size = 3.75 × 3.75 × 3 mm^3^, and total number of volumes = 128. The structural images were scanned with a 3-dimensional (3D) magnetization-prepared rapid gradient-echo (MPRAGE) sequence using the following parameters: TR = 11.864 ms, TE = 4.932 ms, FA = 20°, thickness = 1.4 mm, number of slices = 112, matrix size = 256 × 256, FOV = 152 mm × 152 mm, voxel size = 0.59 × 0.59 × 1.4 mm^3^.

Then, the functional data were preprocessed with resting-state fMRI data processing assistant (DPABI_7.0, http://rfmri.org/DPABI) based on the MATLAB 2013b (https://www.mathworks.com/products/matlab) platform. The first 5 time points were removed, followed by slice-time and motion corrections. The remaining images were normalized to the Montreal Neurological Institute (MNI) space and resampled to a voxel size of 3 × 3 × 3 mm^3^. Then, the data were spatially smoothed using a Gaussian kernel with full width at half maximum (FWHM) of 4 mm × 4 mm × 4 mm. The nuisance variables including 24 motion parameters, white matter signal, cerebrospinal fluid signal, as well as linear trend were regressed out by a general linear mode. Finally, a time-bandpass filter was performed (0.01 Hz < *f* < 0.10 Hz) to eliminate the influence of high-frequency physiological noise and low-frequency drift noise. Participants were excluded if their heads translated more than 3.0 mm or rotated more than 3.0°.

Bilateral OB, PC, and EC were selected as regions of interest (ROIs). The OB was manually segmented within 2-mm MNI space according to the anatomical description in the literature [46]. The MNI coordinates of PC ([−22, 0, −14], [22, 2, −12]) [47] and EC (±26, −1, −33) [48] are from previous literature. A pellet with a radius of 6 mm centered on the coordinates of the seed point serves as the ROIs. ROI-wise analysis method was used to calculate the correlation coefficients among the 6 ROIs using REST (http://restfmri.net/forum/REST V1.8). Finally, the obtained results were converted by Fisher-*z* for statistical analysis.

### 6-OHDA injection

Briefly, mice were anesthetized by intraperitoneal injection of isoflurane gas/oxygen mixture (2%), followed by injection of 1 μl of 6-OHDA (6-hydroxydopamine hydrobromide) hydrochloride (H4281; Sigma, Shanghai, China) solution (dissolved in ice-cold saline solution containing 0.9% NaCl and 0.2 mg/ml l-ascorbic acid from BBI Life Sciences, Shanghai, China) at a concentration of 3 mg/ml or vehicle (0.9% NaCl, 1 μl) in dorsal striatum (AP: +0.50 mm; ML (mediolateral): +1.50 mm; DV (dorsoventral): −3.00 mm) at a rate of 0.2 μl/min [49,50]. The behavioral experiments were conducted 10 d after 6-OHDA injection.

### Behavioral tests

All behavioral experiments were performed during the day. For a week before the experiment, the mice weighed daily and adapted to the environment. The mice were allowed to explore freely in the testing room for 10 min before starting the experiment. All experimental areas were cleaned with 75% ethanol before the tests. Data were collected by the software (ANY-maze, Stoelting, USA).

To check cognitive memory of mice, an olfactory-related working memory test was performed. Cheese (1 cm × 1 cm × 1 cm) was used. One cheese was hidden behind the fixed wooden objects (object A condition), and the other cheese with a metal cover was placed on the top of fixed wooden object (object B condition), which prevents mice from eating the cheese. To eliminate the position preference of mice, position of A and B was variable and 10 cm away from the 2 side walls. The mouse was placed with its back facing the object, and exploration of mouse was recorded by a high-definition camera. Each mouse was subjected to the training session for 10 min every day and sustained for 7 d, then to test session for 10 min. The latency to first exploration of zone A or B and time in zone A or B were quantified.

### Immunohistochemistry

The brain tissue was sectioned into 40-μm-thick slices and stored in the cryoprotectant containing 50% phosphate-buffered saline (PBS), 30% glycol, and 20% glycerol. The slices underwent triple washing with PBS (10 min each) [51]. The brain sections were exposed to the following primary antibodies for 48 h: BOD1 (Sigma Aldrich), CaMKIIα (Abcam, catalog no. ab22609), c-Fos (Synaptic Systems, catalog no. 226003), anti-PCDH21 (Santa Cruz Biotechnology, catalog no. 514574), and 4′,6-diamidino-2-phenylindole (DAPI) (Thermo Fisher Scientific, catalog no. D1306), and then the fluorescence secondary antibodies were hybridized and observed with a confocal microscope (Zeiss LSM 800).

To ascertain retrograde labeling of aPC-projecting and EC-projecting OB M/T cells, coronal sections containing OB were imaged (Zeiss LSM 800). Three OB sections of each mouse were chosen along the anterior–posterior axis, and 2 symmetrical views of each section were selected for each mouse.

### Brain slice preparation

Mice were used after 3 weeks following adeno-associated virus (AAV) injection. The brains were rapidly extracted and placed in ice-cold, oxygenated cutting solution containing the following composition [52]: 75 mM sucrose, 87 mM NaCl, 1.25 mM NaH_2_PO_4_, 2.5 mM KCl, 0.5 mM CaCl_2_, 25 mM NaHCO_3_, 7 mM MgCl_2_, and 25 mM glucose. Slices of 300-μm thickness were then meticulously cut using a vibratome (VT1000S Leica). These slices were subsequently transferred to standard artificial cerebrospinal fluid (ACSF) with the following composition: 124 mM NaCl, 1.25 mM NaH_2_PO_4_, 3 mM KCl, 26 mM NaHCO_3_, 2 mM CaCl_2_, 1 mM MgSO_4_, and 10 mM glucose. After a 30-min incubation period in ACSF at 34 °C, the slices were maintained at room temperature (24 ± 1 °C) for 1 h. The external solutions were completely saturated with a mixture of 5% CO_2_ and 95% O_2_.

### Whole-cell recordings

M/T cells from OB were recorded using a MultiClamp 700B amplifier and 1550A digitizer (Molecular Devices). Neurons expressing EGFP and mCherry were observed with a laser optic microscope equipped with a 40× lens (Olympus). In slice recordings, the electrode resistance ranged from 3.5 to 5.5 MΩ. Neurons were held at −70 mV with a pipette solution [51]. Membrane time constant (Tau), input resistance (*R*_in_), membrane capacitance (C_m_) and APs were recorded as previously reported [53]. Data were analyzed with Clampfit 10 (Molecular Devices) and MATLAB (MathWorks). All drugs and reagents were obtained from Sigma or Tocris.

### Stereotaxic injection

Stereotaxic injection procedures were conducted following previously established methods [54]. For the optogenetic manipulation, pAAV2/9-EF1α-DIO-mCherry-WPRE (AAV-DIO-mCherry, 5.81 × 10^13^ particles/ml) or pAAV2/9-EF1α-DIO-eNpHR3.0-mCherry-WPRE (AAV-DIO-eNpHR3.0-EGFP, 1.27 × 10^13^ particles/ml, Obio Technology, Shanghai, China) was microinfused unilaterally into the ventral OB of 6-week-old *CaMKII*-*Cre* mice.

For the retrograde tracing, AAV2/R-EF1α-EGFP-WPRE-hGHpA (5.60 × 10^12^ particles/ml) and AAV2/R-EF1α-mCherry-WPRE-hGHpA (5.00 × 10^12^ particles/ml, Brain VTA Co. Ltd., Wuhan, China) were microinfused unilaterally into the aPC and EC of 6-week-old *wildtype* mice.

For selective knockout of *Bod1*, pAAV2/9-CAMKIIα-GFP-2A-Cre (4.82 × 10^12^ particles/ml) or pAAV2/9-CAMKIIα-MCS-EGFP-3FLAG (4.77 × 10^12^ particles/ml, Obio Technology, Shanghai, China) was microinfused unilaterally into the ventral OB of 6-week-old *Bod1^f/f^* mice.

The viral vectors were microinfused bilaterally at 200 nl per side, delivered at a rate of 50 nl/min using glass pipettes through a stereotaxic device (RWD Life Science) at the following stereotaxic coordinates: ventral OB (AP: +4.28 mm, ML: ±1.00 mm, DV: −2.25 mm), aPC (AP: +2.10 mm, ML: ±2.00 mm, DV: −4.00 mm), and EC (AP: −3.52 mm; ML: ±3.50 mm; DV: −4.50 mm).

### Optical fiber and electrode implantation

Optical fiber implants were performed as previously [7,23]. A ceramic ferrule with an optical fiber (diameter: 200 μm, numerical aperture (NA): 0.37) was bilaterally implanted, positioning the fiber tip into the aPC and EC following virus injection. Three weeks after implantation, optogenetic inhibition experiments were conducted using a 589-nm yellow laser diode. The laser output at the fiber tip was measured with a Master-9 pulse stimulator (A.M.P.I.) and adjusted to 10 mW before behavioral analysis.

To monitor neuron activity, we implanted the tetrodes into the ventral OB (AP: +4.28 mm, ML: ±1.00 mm, DV: −2.25 mm), aPC (AP: +2.10 mm, ML: ±2.00 mm, DV: −4.00 mm), and EC (AP: −3.52 mm, ML: ±3.50 mm, DV: −4.50 mm) [23]. Each tetrode assembly comprises 8 polyimide-coated nichrome wires connected to a 32-channel electrode interface board (EIB-32, Neuralynx). Following a 3-week implantation period, neural signals during behavioral tests were digitized utilizing the Neuralynx Digital Lynx system through a multiplexing digital headstage. Offline spike sorting was conducted to discriminate single units using the Plexon software. Data analysis was performed using MATLAB 2020a (The MathWorks Inc., Natick, MA, USA).

### Analysis of LFP signals

LFPs were divided into different frequency bands: theta (3 to 12 Hz), beta (15 to 35 Hz), and gamma (40 to 95 Hz), and analyzed using MATLAB scripts [55–58]. PAC is used to evaluate the theta signal-modulated amplitude of the gamma signal, and the color of comodulogram indicates the phase-locked value. The rose diagram shows the phase concentration of spike firing relative to LFP. The spike-LFP phase vector was calculated to represent the locked phase and intensity, and the significance was determined using Rayleigh *z* test.

Wavelet coherence function and cross-spectral function in MATLAB were used to calculate the coherence of electrode pairs with 1,000-Hz sampling rate. Subsequently, data were quantified by the average of all electrode pairs within the OB-aPC, OB-EC, and aPC-EC regions. The Granger causality spectrum between the electrode pairs was calculated at a sampling frequency of 1,000 Hz. For quantification, data were obtained and averaged across OB-aPC and OB-EC electrode pairs.

### Western blotting

Tissue samples from OB were dissected carefully and homogenized with lysis buffer, and then the protein was quantified as previously reported [59]. Primary antibodies included Bod1 (generously provided by Hengyu Fan, Zhejiang University [32]) and β-actin (Multi Sciences, catalog no. ab-008). Immunoreactive proteins were detected with the EZ-ECL Chemiluminescence Detection Kit (Biological Industries).

### Identification of differentially expressed genes

Six PD patient-related databases and 2 PD model mouse-related databases were used to screen the intersection of differentially expressed (DE) genes with a *P* value of <0.05. Six overlapping DE genes were obtained (*BOD1*, *ABHD2*, *APBA1*, *DNAJC5, MFSD6*, and *PEX5L*).

### Quantification and statistical analyses

The sample size (*n*) for each experiment was specified in the respective figure legends. Investigators remained blinded to group allocation and sample identities throughout applicable experiments. Statistical analyses for behavioral and imaging data were conducted using GraphPad Prism 9. Comparisons between 2 groups were assessed using the unpaired 2-sample Student's *t* test. Multiple comparisons were performed using either one-way analysis of variance (ANOVA) (with the Newman–Keuls test) or 2-way ANOVA. Data are presented as mean ± SEM in all figures, with statistical significance denoted as follows: *P < 0.05, **P < 0.01, ***P < 0.001, ****P < 0.0001, ^#^*P* < 0.05, ^##^*P* < 0.01.

## Data Availability

All data required to support the conclusions are presented in the main text and the Supplementary Materials.
