# Supplementary material for: Distinct Olfactory Bulb-Cortex Neural Circuits Coordinate Cognitive Function in Parkinson’s Disease
Source: Research (Wash D C). 2024 Oct 2;7:0484. doi: 10.34133/research.0484 (PMC11445789; doi:10.34133/research.0484)
Supplement: Supplementary 1 — Figs. S1 to S4 [file research.0484.f1.zip › Supplementary materials.docx]

**Supplementary Information for**

**Distinct** **olfactory bulb-cortex neural circuits coordinate cognitive function in Parkinson Disease**

Xing-Feng Mao^1,#^, Shuai-Shuai Wang^2,#^, Zhi-Shen Cai^2^, Wen Lin^2^, Xiu-Xiu Liu^2^, Bei Luo^3^, Xiang Chen^2^, Heng-Yu Fan^4^, Takuya Sasaki^5^, Kohji Fukunaga^5^, Wen-Bin Zhang^3*^, Ying-Mei Lu^1,6*^, Feng Han^2,7,8*^

**Supplementary Figures**

**
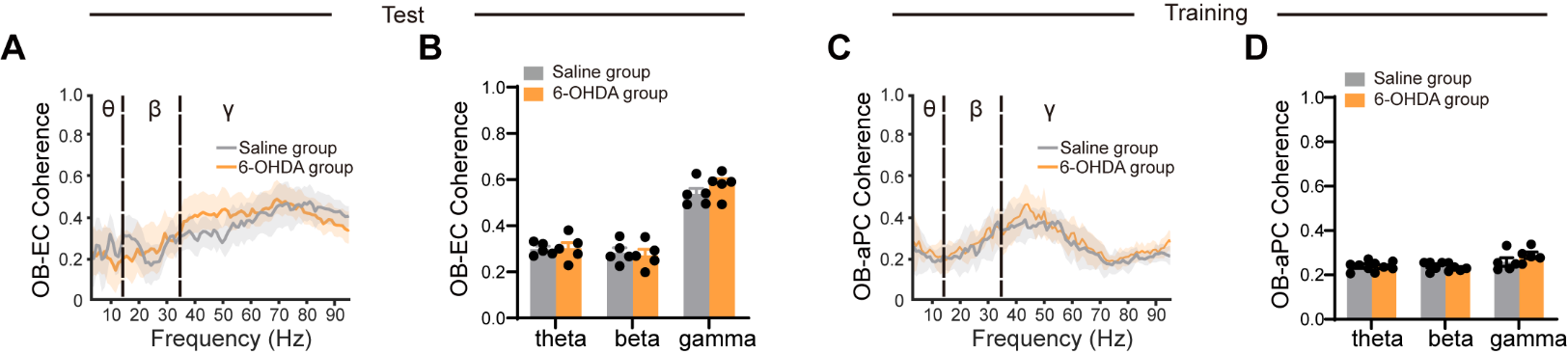
**

**Fig. S1 PD mice showed no significance in OB-aPC coherence during the test session, and OB-EC coherence during the training session.** (A and B) The OB-aPC coherence of LFPs in control and PD mice during the test session (n=5 in each group). (C and D) The OB-EC coherence of LFPs in control and PD mice during the training session (n=5 in each group). Data are presented as mean ± SEM; ns, not significant. Two-way ANOVA for B. Unpaired two-tailed Student’s t test for A.


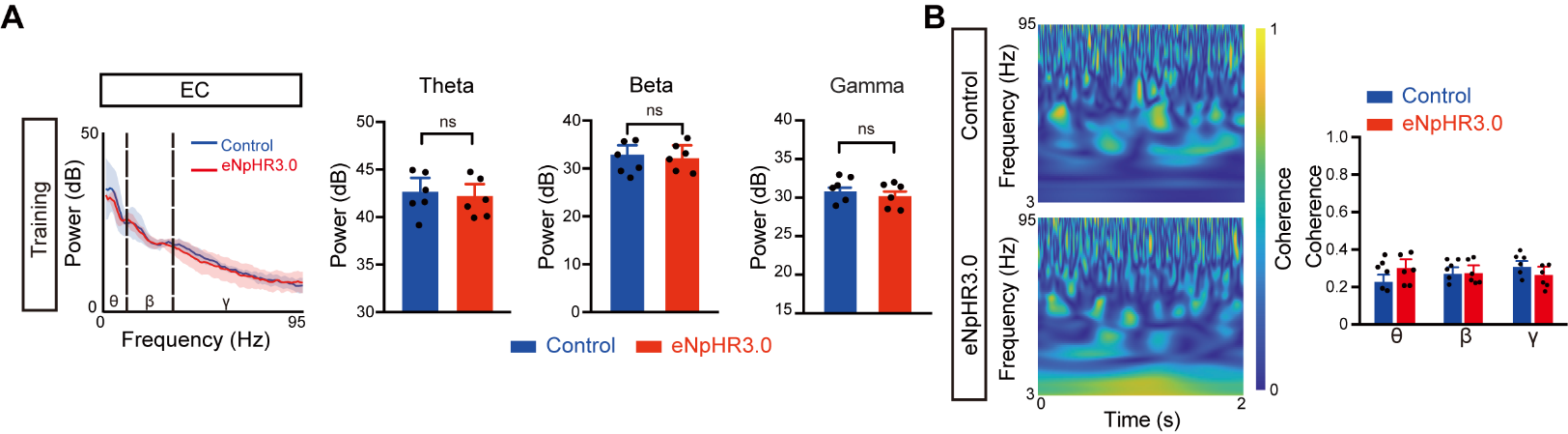


**Fig. S2 Optogenetic inhibition of OB^M/T^→EC circuit had no effects on gamma oscillation and oscillatory coherence in the training session.** (A) Power spectrum of LFP (3–95 Hz) in the EC during the exploration in Zone A in the training session. theta band (θ, 4-12 Hz); beta band (β,13-35 Hz); gamma band (γ, 40-95 Hz) (n=6 in each group). (B) Heatmap and statistical analysis of OB-EC coherence of LFPs in control and optogenetic inhibition mice during the training session (n=6 in each group). Data are presented as mean ± SEM; ns, not significant. Two-way ANOVA for B. Unpaired two-tailed Student’s t test for A.


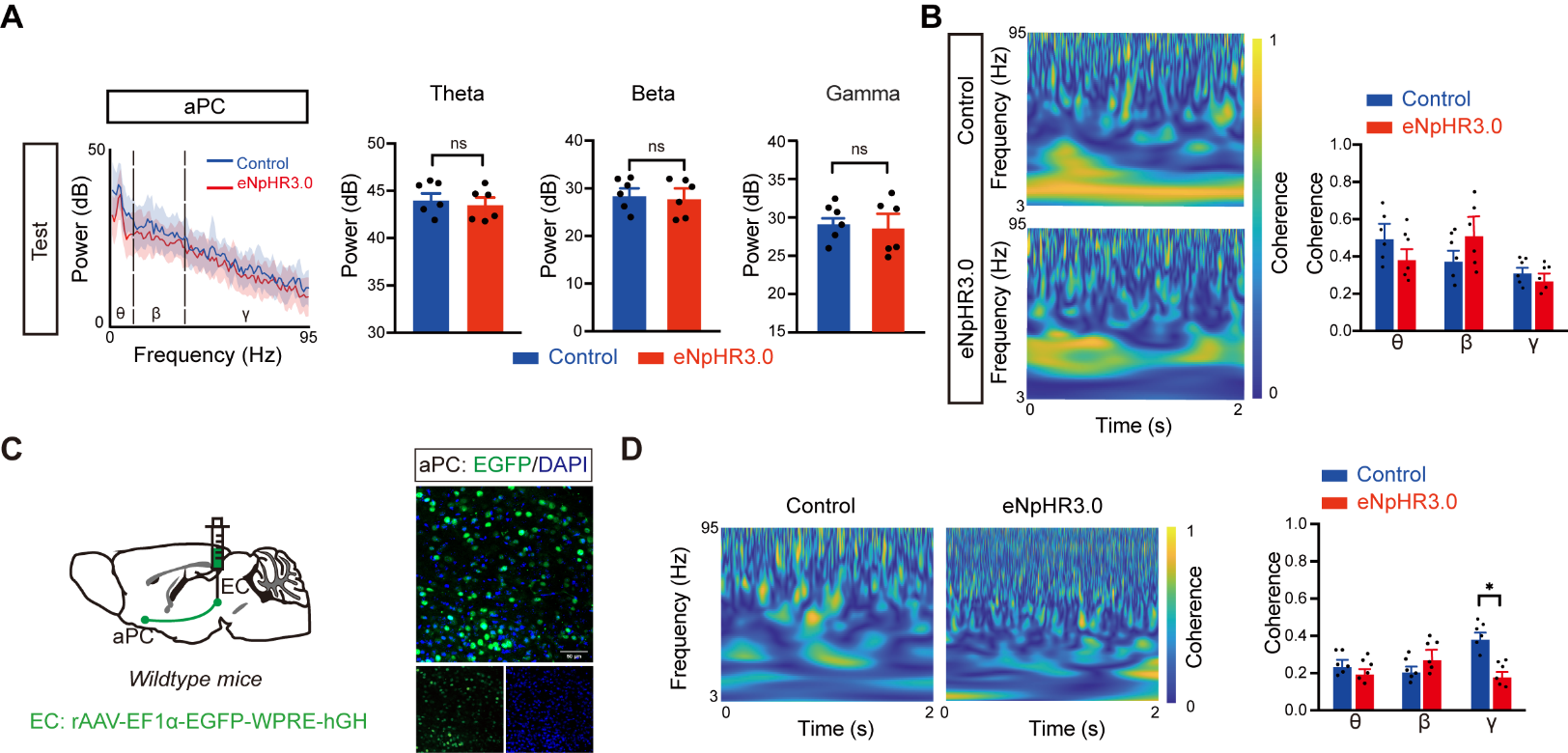


**Fig. S3 Optogenetic inhibition of OB^M/T^→aPC circuit showed no changes in β oscillation and OB-aPC coherence and decreased aPC→EC gamma coherence in the test session.** (A) Power spectrum of LFP (3–95 Hz) in the aPC during exploring the Zone A in the test session (n=6 in each group). (B) Heatmap and quantification of OB-aPC coherence of LFPs in control and optogenetic inhibition mice during the test session (n=6 in each group). (C) Schematic of AAV2/R-EF1α-DIO-EGFP-WPRE-hGHpA injection into EC (left) and histology of EGFP^+^- cells in aPC (right). (D) Heatmap and quantification of aPC-EC coherence of LFPs in control and optogenetic inhibition mice during the test session (n=6 in each group). Data are presented as mean ± SEM; ^*^*P* < 0.05; ns, not significant. Two-way ANOVA for B, D. Unpaired two-tailed Student’s t test for A.


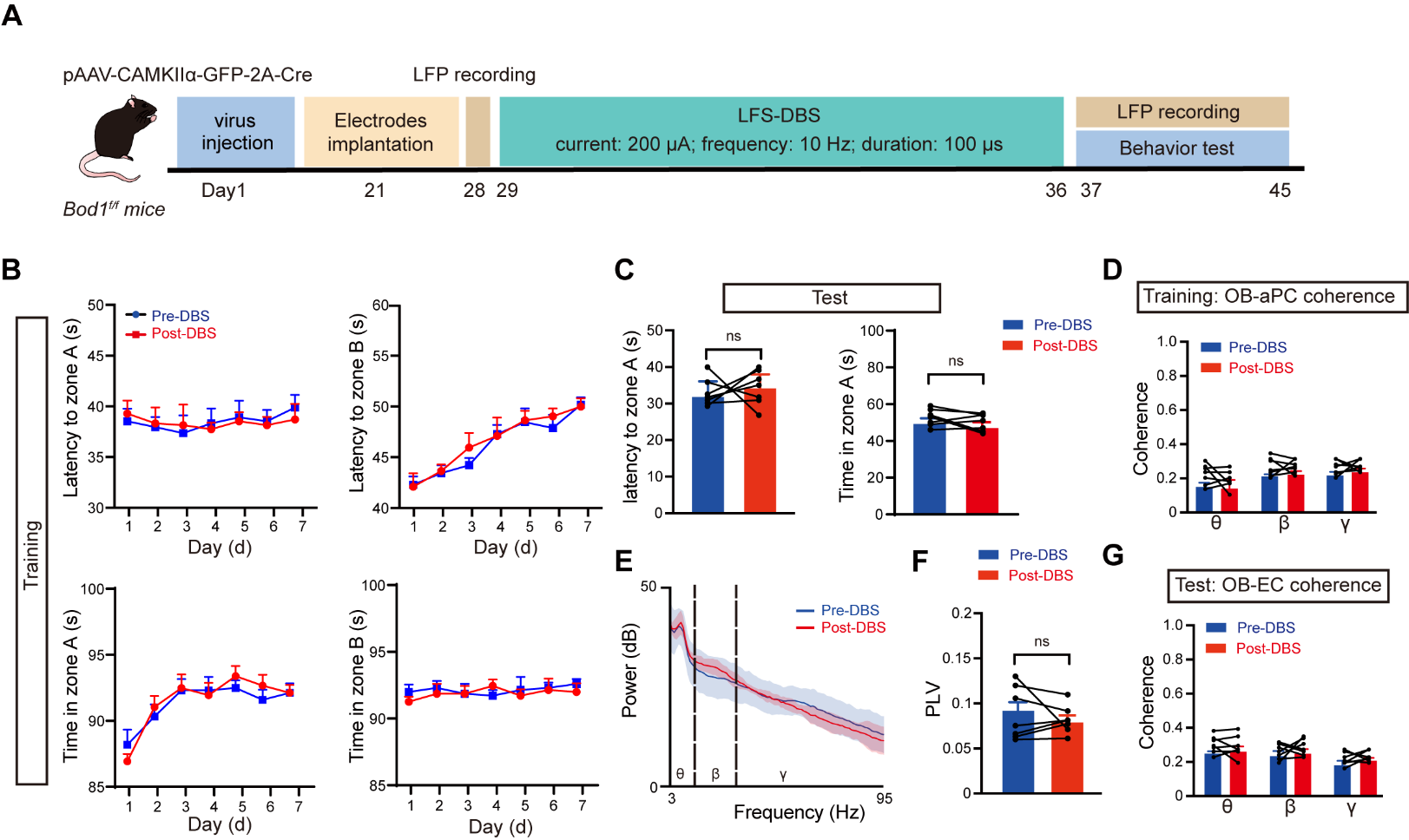


**Fig. S4 LFS treatments had no effect on OB-aPC coherence at beta band and OB-EC coherence at gamma band in** **olfactory-related working memory test.** (A) Schematic of LFS-DBS combined with olfactory-related working memory test. Low-frequency stimulation (LFS) current to OB: 200 μA; frequency: 10 Hz; duration: 100 μs. (B and C) Quantification of latency to Zone A and total time in Zone A and B during the training and test session between pre- and post- DBS in *Bod1*-deficient mice (n=7). (D) The OB-aPC coherence of LFPs in pre- and post-DBS in *Bod1*-deficient mice during the training session (n=7). (E) Power spectrum of LFPs in pre- and post- DBS in *Bod1*-deficient mice (n=7). (F) Quantification of average PAC-modulation index in relatively high-frequency bands coupled to fixed theta bands in pre- and post-LFS DBS in *Bod1*-deficient mice during the test session (n=7). (G) Quantification of the OB-EC coherence of LFPs in pre- and post- DBS in *Bod1*-deficient mice during the test session (n=7). Data are presented as mean ± SEM; ns, not significant. Two-way ANOVA for B, D, G. Paired *t* test for C, F.
